# Changes in Waist Circumference and the Incidence of Type 2 Diabetes in Community-Dwelling Men and Women: The Suita Study

**DOI:** 10.2188/jea.JE20140160

**Published:** 2015-07-05

**Authors:** Yukako Tatsumi, Makoto Watanabe, Michikazu Nakai, Yoshihiro Kokubo, Aya Higashiyama, Kunihiro Nishimura, Takashi Kobayashi, Misa Takegami, Yoko M. Nakao, Takuya Watanabe, Akira Okayama, Tomonori Okamura, Yoshihiro Miyamoto

**Affiliations:** 1 Department of Preventive Medicine and Epidemiology Informatics, National Cerebral and Cardiovascular Center, Suita, Osaka, Japan; 1 国立循環器病研究センター予防医学疫学情報部; 2 Department of Mathematical Health Science, Graduate School of Medicine, Osaka University, Suita, Osaka, Japan; 2 大阪大学大学院総合ヘルスプロモーション科学講座; 3 Department of Preventive Cardiology, National Cerebral and Cardiovascular Center, Suita, Osaka, Japan; 3 国立循環器病研究センター予防健診部; 4 Department of Preventive Medicine and Public Health, Keio University, Tokyo, Japan; 4 慶應義塾大学医学部衛生学公衆衛生学講座

**Keywords:** waist circumference, type 2 diabetes mellitus, prospective cohort study

## Abstract

**Backgrounds:**

The association between weight gain and the incidence of type 2 diabetes is well known. The aim of our study was to investigate the relationship between change in waist circumference (WC) and type 2 diabetes incidence.

**Methods:**

The participants in the Suita Study, a population-based cohort study in an urban area of Japan, underwent a baseline survey between 1989 and 1994 (Exam 1) and were examined at follow-up every 2 years. We performed a 9.3-year cohort study of 946 men and 1327 women with no history of diabetes who underwent Exam 1 and Exam 2 (between 1997 and 1999). Participants were stratified by sex and median WC at Exam 1, and, in each stratum, participants were further classified into three categories by tertile of WC change per year between Exam 1 and Exam 2. Hazard ratios (HRs) and 95% confidence intervals (CIs) for type 2 diabetes incidence were calculated by Cox proportional hazard models. The endpoints were first diagnosis of type 2 diabetes or March 2011.

**Results:**

During follow-up, 287 participants developed type 2 diabetes. In both sexes with median WC or higher, participants in the highest tertile of WC change had a significantly higher risk of developing type 2 diabetes. Multivariable adjusted HRs were 1.84 (95% CI, 1.10–3.08) in men and 2.30 (95% CI, 1.31–4.04) in women. No significant association was observed among participants with WC below median.

**Conclusions:**

Preventing WC gain is important in preventing type 2 diabetes in the Japanese population, especially among individuals with a relatively high WC.

## 要約

**背景：:**

これまで体重増加と2型糖尿病発症の関連については多くの報告があるが、腹囲の増加と2型糖尿病発症の関連についてはほとんど検討されていない。そこで、本研究では日本人集団において腹囲の変化と2型糖尿病発症との関連を明らかにすることを目的とした。

**方法：:**

吹田研究は大阪府吹田市住民から無作為抽出した者を対象としたコホート研究であり、1989年から1994年にベースライン調査（調査Ⅰ）を実施し、その後2年毎に追跡のための健康診断を実施している。本研究では、1997年から1999年の間に追跡のための健康診断（調査Ⅱ）を受診し、糖尿病の既往のない男性946名、女性1327名を2011年3月まで追跡した(平均追跡期間9.3年)。性別に調査Ⅰの腹囲の中央値で層化し、各層において調査Ⅰから調査Ⅱの間の腹囲の変化量（1年あたり）を3分位点で3群[変化量の低い方から順にT1, T2, T3]に分類した。2型糖尿病発症をアウトカムとし、Cox比例ハザードモデルにより、そのハザード比（95%信頼区間）を各層で算出した。

**結果：:**

追跡期間中、287名が2型糖尿病を発症した。腹囲が中央値より高い層において、男女とも、T2群と比較して、T3群で有意な2型糖尿病発症リスクの上昇が認められた。多変量調整後のハザード比は、男性：1.84(1.10-3.08)、女性：2.30 (1.31–4.04)であった。腹囲が中央値より低い層では、男女とも腹囲の変化と2型糖尿病発症リスクの間に有意な関連を認めなかった。

**結論：:**

日本人集団で腹囲増加を予防することは、特に比較的腹囲の大きい集団において、2型糖尿病の発症予防のために重要であることが示唆された。

## INTRODUCTION

The worldwide prevalence of type 2 diabetes is alarmingly high. The International Diabetes Federation (IDF) has reported that the global prevalence of diabetes has reached 8.3% (382 million people), and that the prevalence will be 10% by 2035.^1^ In particular, of IDF regions, the Western pacific region, which includes China, Indonesia, and Japan, has a high prevalence of diabetes (8.6%) and is home to 36% of the total number of people with diabetes in the world.^2^ At the same time, the prevalence of obesity is escalating worldwide. The mean body mass index (BMI) worldwide has increased by 0.4 kg/m^2^ per decade in men and 0.5 kg/m^2^ per decade in women.^3^ Although the prevalence of obesity or overweight in Asia is relatively low compared with other parts of the world, the drastic increase in BMI in Asia is similar to that in other regions. Many studies have reported significant associations between weight gain and the incidence of type 2 diabetes.^4^^–^^10^ Therefore, it is anticipated that this increase in obesity will lead to increased rates of type 2 diabetes.

It is well known that higher waist circumference (WC), as well as higher BMI, is associated with elevated risks of type 2 diabetes.^11^ WC, an index for central obesity, is an important component in the diagnostic criteria for metabolic syndrome.^12^^,^^13^ Because WC changes over the years within the same individual, in addition to assessment of the risk of type 2 diabetes based on WC at a certain point, it would be also useful to consider subsequent WC change. However, there have been just two studies on the association between WC change with the risk of type 2 diabetes, which were conducted among Iranian community residents and American health professionals.^7^^,^^14^ However, percentage of visceral adipose tissue in abdominal fat is likely to differ according to race, so the impact of change in WC on type 2 diabetes could also differ from country to country.^15^ This association remains unknown among the East Asians, who have a relatively low degree of obesity. In addition, it has recently also been reported that WC change does not necessarily correspond to weight change in a Chinese population.^16^

Accordingly, the purpose of this study is to investigate the association between WC change and the incidence of type 2 diabetes in an urban Japanese population, taking into consideration the influence of BMI change.

## METHODS

### Subjects and design

**Figure 1.**
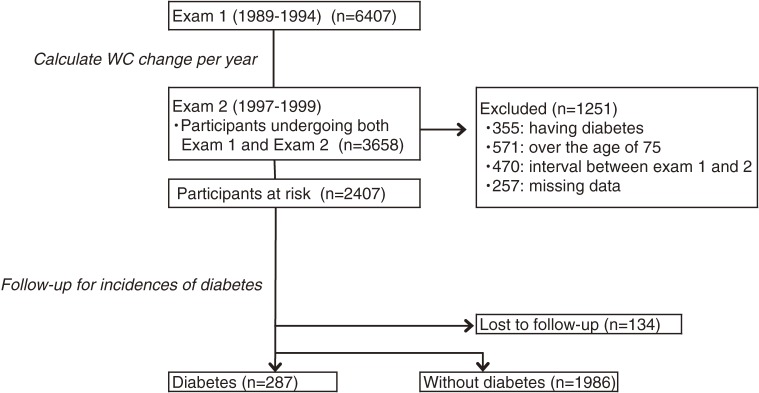
Process of selecting the study participants and overview of analysis of waist circumference (WC) change and the incidence of type 2 diabetes (shown in italics).

The Suita Study, a prospective population-based cohort study in an urban area of Japan, started in 1989. The details of this study have been described elsewhere.^17^^–^^19^ Briefly, 6407 men and women aged 30–83 years underwent a baseline survey at the National Cerebral and Cardiovascular Center (NCVC) between September 1989 and March 1994 (examination 1 [Exam 1]) and visited the NCVC every 2 years for follow-up examinations, including blood sample testing. Of 6407 participants, 3658 underwent the follow-up examination between April 1997 and March 1999 (examination 2 [Exam 2]). Overall, 1251 participants were excluded for the following reasons: (i) having diabetes at Exam 2 (*n* = 355); (ii) age >75 years at Exam 2 (*n* = 571); (iii) the interval between Exam 1 and Exam 2 was <5 years or >9 years (*n* = 470); or (iv) missing data (*n* = 257). In addition, participants who could not be followed-up (*n* = 134) were excluded. The remaining 2273 participants were followed up from Exam 2 to the end of March 2011 (Figure 1). The Institutional Review Board of the NCVC approved this cohort study.

### Data collection

Blood samples were centrifuged immediately upon collection, and a routine blood examination was performed, which included measurement of glucose levels. The Suita Study started to measure HbA_1c_ from Exam 2. The value for HbA_1c_ (%) was estimated as the National Glycohemoglobin Standardization Program equivalent value (%) calculated by the following formula: HbA_1c_ (%) = 1.02 × HbA_1c_ (Japan Diabetes Society, %) + 0.25%.^20^ HbA_1c_ values are presented as percentages and SI units (mmol/mol).

Trained physicians measured blood pressure in triplicate on the right arm after 5 minutes of rest using a standard mercury sphygmomanometer. WC was measured at the umbilical level in a standing position. Participants were wearing light clothing during measurement of height and weight. BMI was calculated as weight (kg) divided by the square of height (m). Public health nurses obtained information on cigarette smoking status (current-smoker, ex-smoker, or non-smoker), alcohol drinking status (current-drinker, ex-drinker, or non-drinker) and medical histories.

### Endpoint determination

Type 2 diabetes was defined as either a fasting (at least 8 hours) plasma glucose level ≥7.0 mmol/L (126 mg/dL), non-fasting plasma glucose level ≥11.1 mmol/L (200 mg/dL), HbA_1c_ ≥6.5% (48 mmol/mol),^21^ or the use of antidiabetic agents. The endpoints of the present study were: (i) first diagnosis of type 2 diabetes, or (ii) March 31, 2011. Individuals not examined during follow-up were censored on the date of their last examination.

### Statistical analysis

Participants were stratified by sex and median WC at Exam 1 and were additionally classified into three categories by tertile of WC change per year between Exam 1 and Exam 2. We calculated age-adjusted WC at Exam 1, Exam 2, and endpoint by sex. In addition, we assessed the correlation of changes in WC and BMI per year between Exam 1 and Exam 2.

Cox proportional hazards regression was used to estimate the adjusted hazard ratios (HRs) and 95% confidence intervals (CIs) for the lowest and highest tertiles, with the middle tertile as the reference group. Model 1 was adjusted for age; model 2 was adjusted for age and WC at Exam 1, HbA_1c_, family history of diabetes, and drinking and smoking status at Exam 2; and model 3 was adjusted for model 2 variables, BMI at Exam 1, and BMI change as a continuous variable. In an additional model, HRs and 95% CIs were adjusted for model 2 variables and estimated without stratification by WC at Exam 1, with the participants falling into both the WC below median and the tertile 2 in WC change groups being set as the reference group. Interactions between WC at Exam 1 (below median or ≥median) and WC change (tertiles) were tested by adding the interaction term to the model 2. All data were analyzed using SPSS statistical software (Version 20.0J; SPSS Japan Inc., Tokyo, Japan). All reported *P*-values are two-tailed; *P* < 0.05 was considered statistically significant.

## RESULTS

**Figure 2.**
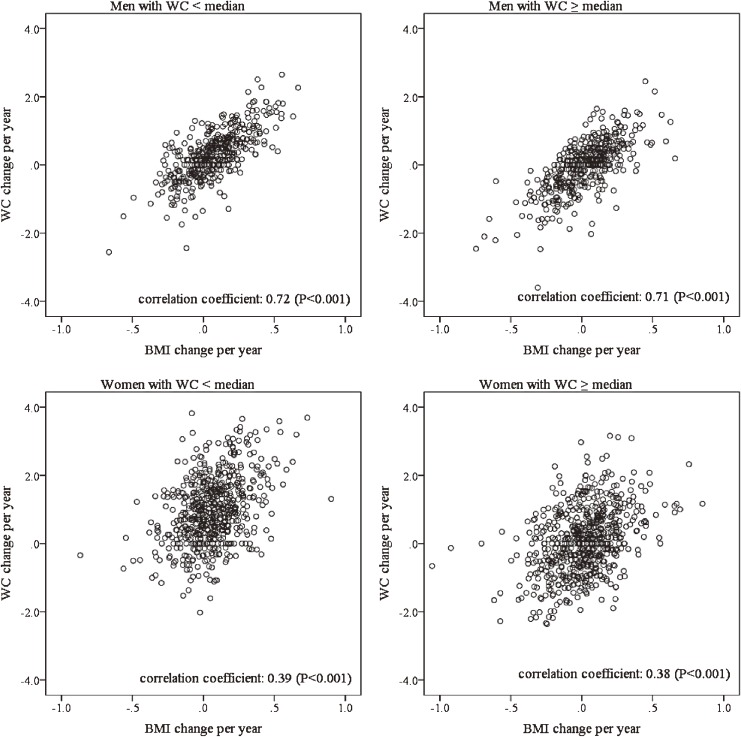
Scatter plots of waist circumference (WC) change and body mass index (BMI) change between examination 1 and examination 2 by sex and WC.

The median (interquartile range) WCs at Exam 1 were 82.0 (77.0–87.0) cm in men and 75.0 (69.0–82.0) cm in women. The mean (standard deviation) interval between Exam 1 and Exam 2 was 6.8 (0.9) years. Table 1 shows characteristics at Exam 2 for men and women. WC change was considerably larger in women than men (0.51 and 0.17 cm/year, respectively). Table 2 shows age-adjusted WC at Exam 1, Exam 2, and at the endpoint examination. In the lowest tertile of WC change, WC increased from Exam 2 to the endpoint examination, regardless of sex and WC strata (WC <median or WC ≥median). Conversely, the change in WC from Exam 2 to endpoint examination in the highest tertile was stable. Figure 2 shows scatter plots of WC change and BMI change between Exam 1 and Exam 2. The correlation coefficients with a WC below the median and at the median or higher were 0.72 and 0.71 in men, respectively, and 0.39 and 0.38 in women, respectively (all *P* < 0.001).

**Table 1.** Characteristics of subjects at examination 2 (*n* = 2273)

	Men	Women
*n*	946	1327
Age, years	58.8 (10.2)	57.6 (9.7)
WC	83.5 (7.9)	79.7 (8.6)
WC change, cm/year	0.17 (0.74)	0.51 (1.05)
BMI, kg/m^2^	23.2 (2.8)	22.2 (2.9)
HbA_1c_, %, mmol/mol	5.6 (0.3), 38 (3.3)	5.6 (0.3), 38 (3.3)
Plasma glucose level, mmol/L	5.3 (0.5)	5.1 (0.5)
Systolic blood pressure, mm Hg	126.4 (18.0)	125.3 (18.9)
Diastolic blood pressure, mm Hg	80.8 (10.7)	78.6 (10.5)
Hypertension, *n* (%)	341 (36.0)	406 (30.6)
Total cholesterol, mmol/L	5.26 (0.81)	5.60 (0.85)
Hypercholesterolemia, *n* (%)	302 (31.9)	644 (48.5)
Family history of diabetes, *n* (%)	90 (9.5)	148 (11.2)
Smoking status, *n* (%)		
Current	372 (39.3)	100 (7.5)
Former	296 (31.3)	47 (3.5)
Never	278 (29.4)	1180 (88.9)
Drinking status, *n* (%)		
Current	690 (72.9)	411 (31.0)
Former	23 (2.4)	9 (0.7)
Never	233 (24.6)	907 (68.3)

BMI, body mass index; WC, waist circumference.

Continuous data are shown as mean (standard deviation).

**Table 2.** Waist circumference and BMI adjusted by age at Exam 1, Exam 2, and endpoint examination (*n* = 2273)

	WC change		

	tertile 1	tertile 2	tertile 3
Men with WC <median^a^			
Range of WC change, cm/year	−2.556 to 0.000	0.117 to 0.552	0.558 to 2.648
Age at examination 2	59.2 (10.6)	57.0 (10.0)	57.1 (10.6)
WC, Exam 1/Exam 2/endpoint	76.9/74.2/77.6	76.3/78.4/80.2	75.9/82.9/83.9
BMI, Exam 1/Exam 2/endpoint	20.9/20.5/20.8	21.3/21.7/21.7	21.2/22.6/22.7
Men with WC ≥median^a^			
Range of WC change, cm/year	−3.598 to −0.265	−0.263 to 0.296	0.304 to 2.454
Age at examination 2	60.9 (10.4)	60.0 (8.8)	58.2 (10.1)
WC, Exam 1/Exam 2/endpoint	90.1/84.7/87.4	88.3/88.6/89.4	87.3/92.1/92.6
BMI, Exam 1/Exam 2/endpoint	24.8/23.8/23.8	24.6/24.8/24.6	24.7/25.8/25.7
Women with WC <median^a^			
Range of WC change, cm/year	−2.023 to 0.506	0.509 to 1.378	1.380 to 3.820
Age at examination 2	53.5 (9.8)	55.0 (10.3)	56.9 (8.9)
WC, Exam 1/Exam 2/endpoint	69.5/69.3/73.3	67.7/74.4/77.3	67.5/81.7/81.7
BMI, Exam 1/Exam 2/endpoint	19.7/20.3/21.2	19.6/20.7/22.2	19.7/20.8/21.9
Women with WC ≥median^a^			
Range of WC change, cm/year	−2.363 to −0.341	−0.334 to 0.463	0.472 to 3.160
Age at examination 2	60.0 (9.6)	59.5 (9.3)	60.1 (8.7)
WC, Exam 1/Exam 2/endpoint	85.3/79.4/82.9	83.4/83.8/84.8	80.8/88.4/88.5
BMI, Exam 1/Exam 2/endpoint	23.0/23.4/23.9	22.5/23.5/24.6	22.6/23.1/24.2

BMI, body mass index; WC, waist circumference.

^a^Medians of waist circumference at examination 1 were 82.0 cm in men and 75.0 cm in women. Ages are shown as mean (standard deviation).

During the follow-up periods (mean 9.3 [3.5] years), 287 participants developed type 2 diabetes (Figure 1). Table 3 shows multivariable adjusted HRs for incidences of type 2 diabetes according to WC change tertile. Among participants with the median WC or higher, the highest tertile had significantly higher risk for the incidence of type 2 diabetes in both sexes in model 2 (HR 1.84; 95% CI, 1.10–3.08 in men and HR 2.30; 95% CI, 1.31–4.04 in women). The lowest tertile did not have a significantly lower risk for the incidence of type 2 diabetes in either sex (HR 1.27; 95% CI, 0.73–2.21 in men and HR 1.13; 95% CI, 0.59–2.18 in women). Among participants with WC below the median, there was no significant association between WC change and the incidence of type 2 diabetes in either sex. Interactions between WC at Exam 1 (below median or ≥median) and WC change (tertiles) was not significant in men (*P* = 0.395) but was significant in women (*P* = 0.011).

**Table 3.** Multivariable adjusted hazard ratios for the incidence of type 2 diabetes according to change in waist circumference (*n* = 2273)

	Cases/*n*	IR^a^	HRs (95% CIs)			

			Model 1	Model 2	Model 3	Additional model
Men with WC <median^b^						
tertile 1	25/169	16.6	1.06 (0.59–1.90)	1.19 (0.66–2.16)	1.01 (0.52–1.96)	1.25 (0.69–2.25)
tertile 2	21/148	15.5	ref	ref	ref	ref
tertile 3	32/158	22.3	1.44 (0.83–2.49)	1.25 (0.71–2.22)	1.34 (0.73–2.46)	1.36 (0.77–2.38)
Men with WC ≥median^b^						
tertile 1	29/157	20.0	1.16 (0.68–1.99)	1.27 (0.73–2.21)	1.40 (0.75–2.58)	1.50 (0.84–2.68)
tertile 2	24/157	16.8	ref	ref	ref	1.18 (0.65–2.15)
tertile 3	39/157	29.3	1.80 (1.08–3.01)	1.84 (1.10–3.08)	1.72 (0.98–3.02)	2.22 (1.28–3.83)
Women with WC <median^b^						
tertile 1	16/213	7.9	1.70 (0.77–3.75)	1.38 (0.60–3.20)	1.98 (0.80–4.91)	1.67 (0.75–3.69)
tertile 2	10/213	4.7	ref	ref	ref	ref
tertile 3	10/213	4.7	0.93 (0.39–2.25)	0.70 (0.28–1.74)	0.52 (0.20–1.38)	0.83 (0.35–2.23)
Women with WC ≥median^b^						
tertile 1	19/229	8.6	0.90 (0.48–1.69)	1.13 (0.59–2.18)	1.24 (0.63–2.44)	1.56 (0.72–3.39)
tertile 2	20/229	9.4	ref	ref	ref	1.24 (0.57–2.69)
tertile 3	42/230	19.9	2.14 (1.26–3.65)	2.30 (1.31–4.04)	2.07 (1.13–3.79)	2.45 (1.22–4.94)

CI, confidence interval; HR, hazard ratio; Ref, reference group; WC, waist circumference.

Model 1: Adjusted by age; Model 2: Adjusted by age, HbA1c, family history of diabetes, smoking and drinking status at examination 2, and WC at examination 1; Model 3: Adjusted by model 2 variables, body mass index at examination 1, and change in body mass index as continuous variables; Additional Model: Without stratification by median of WC at exam 1 and adjusted by model 2 variables except for WC at examination 1; ^a^Incidence rates/1000 person-years; ^b^Medians of waist circumference were 82.0 cm in men and 75.0 cm in women at examination 1.

Even after adjustment of BMI at Exam 1 and BMI change (model 3), these results did not change much, although the HR of the highest tertile of WC change among men with WC median or higher was borderline significant (HR 1.72; 95% CI, 0.98–3.02). In model 3, the HRs for BMI change (per 1.0 kg/m^2^/year) were 2.11 (95% CI, 0.49–9.16) in men with the median WC or higher and the highest tertile of WC change and 0.93 (95% CI, 0.31–2.82) in women. In the additional model, HRs of the highest tertile of WC change significantly increased among both men and women with the median WC at Exam 1 or higher.

## DISCUSSION

The present study demonstrates that, among participants with relatively high WC and regardless of sex, WC gain for 5–9 years was significantly associated with an elevated risk of incidence of type 2 diabetes for almost 10 years following WC gain, after adjustment for baseline HbA_1c_. On the other hand, WC loss was not associated with a decreased risk of incidence of type 2 diabetes. No significant association between WC change and the incidence of type 2 diabetes was observed among individuals with relatively low WC.

To our knowledge, only two studies have reported the association between WC change and type 2 diabetes. Hadaegh et al reported that WC change during 6 years of follow-up was positively associated with the elevated risk of type 2 diabetes among 4029 community residents in Iran.^14^ Odds ratios were 1.6 in men and 1.5 in women per 1 standard deviation increase in WC change (5.2 cm in men and 7.7 cm in women). Koh-Banerjee et al assessed the influence of WC change in 9 years on type 2 diabetes incidences for 4 years after WC change among 22 171 male health professionals in the United States.^7^ They demonstrated that men with WC gain ≥14.6 cm (1.6 cm/year) had a higher risk of developing type 2 diabetes than men who had a stable WC (relative risk 2.4; 95% CI, 1.5–3.7). Participants in these studies had higher average WC than those in the present study.^14^^,^^22^ Because we also observed results similar to previous studies among participants with relatively high WC, the present results would be likely to support the previous ones, although it is difficult to compare the results between the studies because of different observation periods for WC change and ethnicities of participants.

The additional model using the six combined categories of initial WC and subsequent WC change also showed similar results. Compared to individuals with relatively low initial WC levels and subsequent mild WC gain (tertile 2), regardless of sex, WC gain was significantly associated with increased risk of type 2 diabetes only among individuals with relatively high WC levels, while no significant association was observed in other categories. This indirectly suggests that it might be important to combine WC at a certain point and subsequent WC change for estimation of risk of diabetes.

However, an observational study has reported that WC loss was not associated with a decreased risk of type 2 diabetes incidence.^7^ In the present study, the effect of WC loss among participants with relatively high WC was not observed, although it is not clear whether WC loss was caused by participants’ effort, such as lifestyle changes. Among individuals in the lowest tertile whose WC decreased from Exam 1 to Exam 2 (a 5.4-cm decrease in men with relatively high WC and a 5.9-cm decrease women with relatively high WC), WC increased after Exam 2 (a 2.7-cm increase in men with a relatively high WC and a 2.5-cm increase in women with relatively high WC) (Table 2). In other words, WC showed a U-shaped change from Exam 1 to the endpoint examination, and it can be inferred that the risk of type 2 diabetes might not decrease merely because of WC gain after Exam 2.

Koh-Banerjee et al reported that men with WC gain had a higher risk for type 2 diabetes incidence after adjustment for weight change.^7^ Similarly, in the present study, WC change was associated with the incidence of type 2 diabetes almost independently of BMI change among both men and women with relatively high WC levels, although this relationship was more evident in women. On the other hand, BMI change was not associated at all. The present study suggests that WC change should be considered prior to weight change to estimate the risk of type 2 diabetes, especially in Japanese women. In addition, the results of the correlation analyses showed that WC change was much more strongly correlated with BMI change in men than women. This difference in correlation could be involved in the sex difference. Because body fat distribution differs considerably between age groups, sexes, and ethnicities,^23^^,^^24^ it would be necessary to take these factors into consideration when combining WC and BMI to estimate the risk of type 2 diabetes.

The present study has several limitations. First, 62.0% of participants did not undergo Exam 2 under fasting conditions (≥8 hours), although almost all underwent Exam 1 under fasting conditions. However, there was no significant difference in fasting status among the tertiles of WC change regardless of sex and WC strata, and the time after a meal was ≥5 hours in 87% of participants. Such random misclassifications by measurement error might lead to underestimation of the real relationship (toward the null) between WC changes and the risk of diabetes. Second, the correlation coefficients between change in WC and BMI were high in men (0.71–0.72), so the presence of co-linearity might influence the adjusted HRs in model 3 of Cox proportional hazards regression. However, since the HRs did not change much after adjustment for BMI changes, we think the influence of co-linearity was likely to be limited. Third, single assessment of WC change may lead to underestimation of the relationship between WC change and type 2 diabetes incidences due to regression dilution bias.^25^

In conclusion, WC gain was significantly related to an increased risk of type 2 diabetes in both sexes with a higher WC. In terms of diabetes prevention, it is important to avoid WC gain, especially among men and women with relatively high WC. In addition, assessing WC change may be important than assessing BMI change in estimating the risk of type 2 diabetes, especially in the Japanese women.

## ONLINE ONLY MATERIAL

Abstract in Japanese.
