# Supplementary material for: Changes in Waist Circumference and the Incidence of Type 2 Diabetes in Community-Dwelling Men and Women: The Suita Study
Source: J Epidemiol. 2015 Jul 5;25(7):489–95. doi: 10.2188/jea.JE20140160 (PMC4483375; doi:10.2188/jea.JE20140160)
Supplement: Abstract in Japanese. [file je-25-489-s001.pdf]

都市部地域住民における腹囲の変化と 2 型糖尿病発症の関連：吹田研究

辰巳友佳子[1,2]、渡邉至[3]、中井陸運[1]、小久保喜弘[3]、東山綾[1]、西村邦宏[1]、小林貴[3]、竹上未紗[1]、中尾葉子[1]、渡邊琢也[3]、岡山明[3]、岡村智教[4]、宮本恵宏[1,3]
1. 国立循環器病研究センター予防医学疫学情報部
2. 大阪大学大学院総合ヘルスプロモーション科学講座
3. 国立循環器病研究センター予防健診部
4. 慶應義塾大学医学部衛生学公衆衛生学講座

要約
背景： これまで体重増加と 2 型糖尿病発症の関連については多くの報告があるが、腹囲の増加と 2 型糖尿病発症の関連についてはほとんど検討されていない。そこで、本研究では日本人集団において腹囲の変化と 2 型糖尿病発症との関連を明らかにすることを目的とした。
方法： 吹田研究は大阪府吹田市住民から無作為抽出した者を対象としたコホート研究であり、1989 年から 1994 年にベースライン調査（調査Ⅰ）を実施し、その後 2 年毎に追跡のための健康診断を実施している。本研究では、1997 年から 1999 年の間に追跡のための健康診断（調査Ⅱ）を受診し、糖尿病の既往のない男性 946 名、女性 1327 名を 2011 年 3 月まで追跡した（平均追跡期間 9.3 年）。性別に調査Ⅰの腹囲の中央値で層化し、各層において調査Ⅰから調査Ⅱの間の腹囲の変化量（1 年あたり）を 3 分位点で 3 群[変化量の低い方から順に T1, T2, T3]に分類した。2 型糖尿病発症をアウトカムとし、Cox 比例ハザードモデルにより、そのハザード比（95%信頼区間）を各層で算出した。
結果： 追跡期間中、287 名が 2 型糖尿病を発症した。腹囲が中央値より高い層において、男女とも、T2 群と比較して、T3 群で有意な 2 型糖尿病発症リスクの上昇が認められた。多変量調整後のハザード比は、男性：1.84(1.10-3.08)、女性：2.30（1.31-4.04)であった。腹囲が中央値より低い層では、男女とも腹囲の変化と 2 型糖尿病発症リスクの間に有意な関連を認めなかった。
結論： 日本人集団で腹囲増加を予防することは、特に比較的腹囲の大きい集団において、2 型糖尿病の発症予防のために重要であることが示唆された。

キーワード：腹囲、2 型糖尿病、前向きコホート研究
